# Fluorescence-detected linear dichroism imaging in a re-scan confocal microscope equipped with differential polarization attachment

**DOI:** 10.1007/s00249-019-01365-4

**Published:** 2019-04-13

**Authors:** Gabor Steinbach, David Nagy, Gábor Sipka, Erik Manders, Győző Garab, László Zimányi

**Affiliations:** 1 Institute of Biophysics, Biological Research Centre, Hungarian Academy of Sciences, Temesvári krt. 62, Szeged, 6726 Hungary; 2 Institute of Plant Biology, Biological Research Centre, Hungarian Academy of Sciences, Temesvári krt. 62, Szeged, 6726 Hungary; 3 Confocal.nl, Sciencepark 406, 1098 XH Amsterdam, The Netherlands; 4 Biofotonika Research and Development Ltd., Dózsa u. 7., Szeged, 6720 Hungary

**Keywords:** Anisotropy, DP-LSM, Fluorescence-detected linear dichroism, RCM

## Abstract

Confocal laser scanning microscopy is probably the most widely used and one of the most powerful techniques in basic biology, medicine and material sciences that is employed to elucidate the architecture of complex cellular structures and molecular macro-assemblies. It has recently been shown that the information content, signal-to-noise ratio and resolution of such microscopes (LSMs) can be improved significantly by adding different attachments or modifying their design, while retaining their user-friendly features and relatively moderate costs. Differential polarization (DP) attachments, using high-frequency modulation/demodulation circuits, have made LSMs capable of high-precision 2D and 3D mapping of the anisotropy of microscopic samples—without interfering with their ‘conventional’ fluorescence or transmission imaging (Steinbach et al. in Methods Appl Fluoresc 2:015005, 2014). The resolution and the quality of fluorescence imaging have been enhanced in the recently constructed Re-scan confocal microscopy (RCM) (De Luca et al. in Biomed Opt Express 4:2644–2656, 2013). In this work, we developed the RCM technique further, by adding a DP-attachment modulating the exciting laser beam via a liquid crystal (LC) retarder synchronized with the data acquisition system; by this means, and with the aid of a software, fluorescence-detected linear dichroism (FDLD), characteristic of the anisotropic molecular organization of the sample, could be recorded in parallel with the confocal fluorescence imaging. For demonstration, we show FDLD images of a plant cell wall (*Ginkgo biloba*) stained with Etzold’s staining solution.

**Electronic supplementary material:**

The online version of this article (10.1007/s00249-019-01365-4) contains supplementary material, which is available to authorized users.

## Introduction

To understand the molecular architecture of complex, highly organized molecular macro-assemblies is crucial for basic biological research. Differential polarization (DP) spectroscopy techniques provide unique and important information about highly anisotropic molecular macro-assemblies (Tinoco et al. 1987). The ability of the biological structures and intelligent materials to modify the polarization of the incident light is described by the 4-by-4 transformation Mueller matrix (Mueller 1948). This matrix describes the light–matter interactions, which are determined at the molecular level, and thus carry detailed physical information about the organization of the sample. All but one (the absorbance, *M*_11_) of the 16 elements describes polarization of the incident or transmitted light. Although in complex materials all 16 elements are independent and carry physical information on the molecular organization of the sample (Tinoco et al. 1987); in practice, the measurements are confined to a few easy-to-determine quantities, such as linear dichroism (LD, *M*_12_ and *M*_13_) or circular dichroism (CD, *M*_14_).

To obtain information about anisotropy at the microscopic level, e.g. on tissues, single cells or organelles and single molecules, it is important to adapt DP-measurements to optical microscopy. In DP imaging, a difference image is obtained using (usually) two orthogonal polarizations, and the resulting image is a two-dimensional map of the anisotropy of the sample:

${\text{DP}} = \frac{{I_{1} - I_{2} }}{{\bar{I}}},$ where *I*_1_ and *I*_2_ are intensities for the two orthogonal polarization states (1 and 2) and $\bar{I}$ is the average of the total intensity used for normalization.

High-frequency polarization modulation of the measuring beam and demodulation of the detected signal, combined with scanning-stage, allowed the imaging of anisotropy in confocal transmission mode (Mickols et al. 1985); confocal LD and CD images have revealed the existence of chiral macrodomains in granal chloroplasts (Finzi et al. 1989). Similar scheme was employed in the modification of a laser scanning microscope (LSM) in which LD could be imaged (non-confocally) with high precision on thin samples (Gupta et al. 1994). However, the commercially available LSMs offer confocal imaging, thereby yielding much better resolution and contrast than non-confocal imaging, in fluorescence and reflection regimes. Hence, to map anisotropy in confocal mode, DP imaging was extended to fluorescence regime (Garab et al. 2005a), with different DP-attachments and software designed to be compatible with the optics and the data acquisition of the microscope (Steinbach et al. 2014). The DP-LSMs constructed in our laboratory, in addition to LD, CD and birefringence imaging (in non-confocal, transmission mode), are capable of imaging confocally fluorescence-detected linear and circular dichroism (FDLD and FDCD) as well as the anisotropy and the degree of polarization of the fluorescence emission (*r* and *P*, respectively, using non-polarized and polarized excitation) (Garab et al. 2005a; Steinbach et al. 2009, 2014). All these parameters carry unique information about the anisotropic molecular architecture of the samples: FDLD and *r* provide information about the alignment of absorption and emission dipoles, respectively; FDCD about short-range excitonic interactions and long-range chiral order of fluorophores in the sample; *P*, reflecting depolarization of the emission, depends on energy transfer processes and/or molecular motions during the fluorescence lifetime.

The DP-LSM technique has been used successfully on a variety of preparations, including knock-out Drosophila embryos (Gorjánácz et al. 2006), human T lymphoma and B lymphoblast cells (Gombos et al. 2008; Steinbach et al. 2014), aggregated amyloid filaments (Makovitzky 2003; Steinbach et al. 2011), isolated thylakoid membranes, and natural and artificial light-harvesting complexes (Steinbach et al. 2005; Chappaz-Gillot et al. 2012). The DP-measurements provide information about the anisotropic structures of biological objects that is crucial in micromanipulation with polarized light (Garab et al. 2005b).

The pixel-by-pixel modulation method of DP-LSMs is a suitable technique for living cells/tissues; it avoids possible artefacts due to the displacement of the sample during the acquisition process. The recent focus of the research using DP imaging was the organization of the cellulose fibrils of the cell wall in higher plants. Previous studies performed by rotated samples and sequential imaging (Verbelen and Kerstens 2000; Kerstens and Verbelen 2003) and using the first-generation DP-LSM technique (Steinbach et al. 2008), proved the anisotropic organization of the cellulose macromolecules in the cell wall. Further works investigated the differences in FDLD between different plant materials (hard wood, soft wood and maize) and environmental conditions, and mechanobiological aspects (Djikanović et al. 2016; Savić et al. 2016; Radosavljević et al. 2017). The results of these works provided new data for comparison of the cell wall properties that may be important for the selection of appropriate plant and growth conditions for possible applications as a source of biomass.

Re-scan confocal microscopy (RCM) has been invented recently as a special attachment for fluorescence microscopes providing confocal images. The technique is based on the standard confocal principle using laser beams for excitation of the fluorophores, but it has an essentially different image acquisition technique. The imaging light path of the RCM is extended with a second (re-)scanning mirror unit that projects the acquired light on the camera chip as an image rather than on a single photodetector (photodiode or photomultiplier tube) (De Luca et al. 2013). RCM thus establishes a pixel-by-pixel correspondence between the scanned sample area and the (re-)scanned detector area and improves the lateral resolution by a factor of 1.4 and the axial resolution (capability of the optical sectioning) by 15% without using any post-acquisition image processing algorithm. Furthermore, the signal-to-noise ratio using the RCM is a factor of 2 higher than in standard PMT-based confocal laser scanning microscopes (CLSMs), due to the use of highly sensitive modern cameras (De Luca et al. 2017, and see Supplementary Figure 1). For these advantageous features, RCM is useful for biological applications, where the combination of high resolution and high sensitivity is required; it would thus be of interest to make this technique capable of providing information about the anisotropic molecular organization of the sample, parallel with the fluorescence imaging. Wide-field, confocal and multiphoton systems extended with polarization sensitive attachments were used by several studies (Lazar et al. 2011; Wang et al. 2013; Hafi et al. 2014; Mazumder et al. 2017; Loison et al. 2018), but uniting the breakthrough in resolution and mapping polarization properties improves our understanding of ordered molecular structures. The focus of our current study described in this paper is building a DP package (hardware and software elements) to extend the capacity of the RCM unit with mapping structural anisotropy by FDLD, FDCD, *P* and *r* imaging, using modulated polarized excitation or emission. As a first step in this direction, we have equipped our RCM unit with a DP-attachment, which, in combination with suitable software, allows the recording of confocal FDLD images, without perturbing the operation of RCM. As a proof-of-concept, we show the FDLD image of the cell wall structure of *Ginkgo biloba* tissue.

## Materials and methods

### Base DP system

**Fig. 1:**
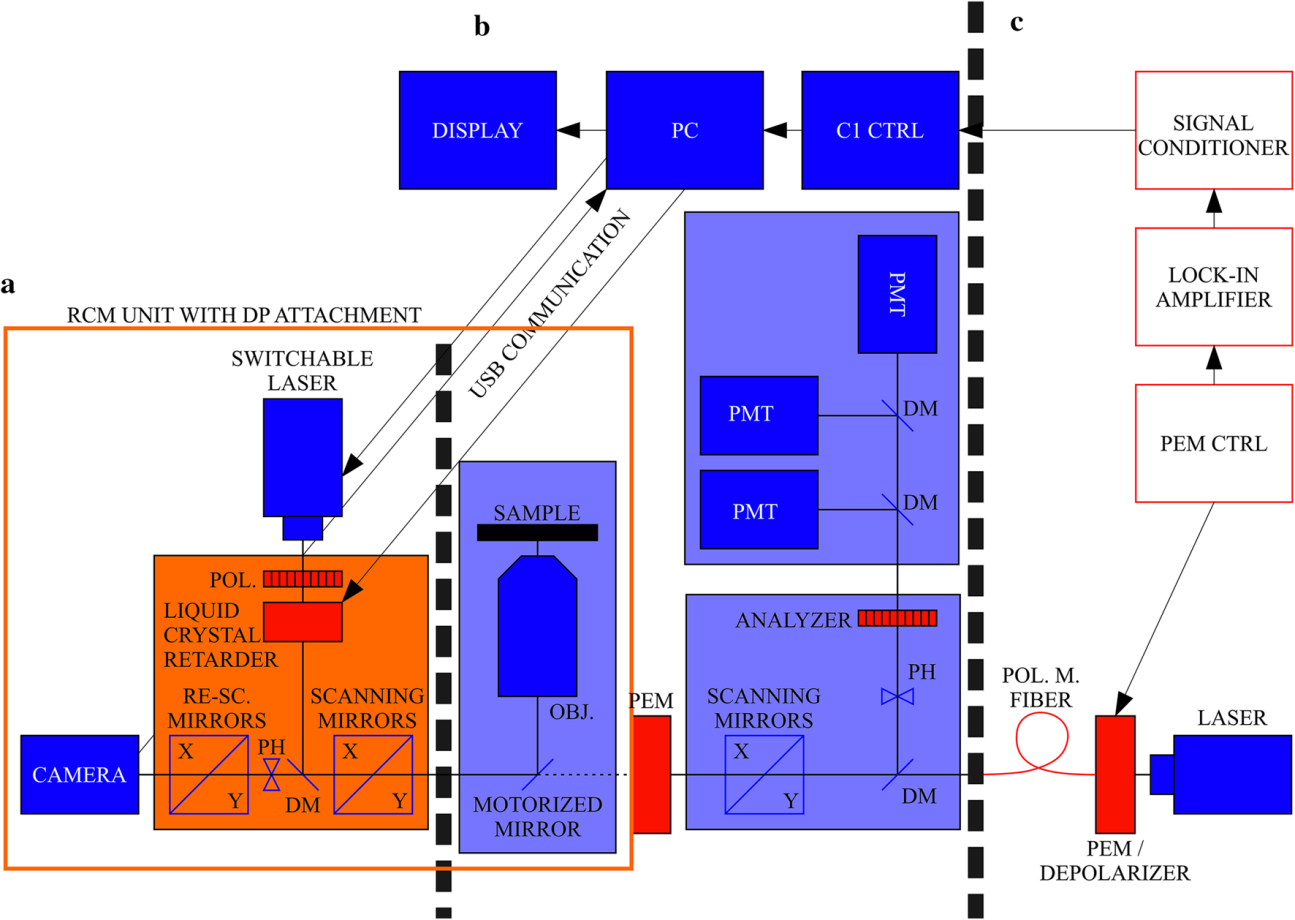
(**a**) RCM unit equipped with the liquid crystal-based DP unit (*POL* polarizer, *PH* pinhole). (**b**) The original Nikon C1 microscope (*OBJ* objective, *DM* dichroic mirror, *PH* pinhole). (**c**) High-frequency differential polarization attachment for the C1 unit, in which polarization of the excitation/emission is modulated using photoelastic modulators (PEMs) and phase-sensitive detection by a lock-in amplifier

The major units of the DP system are shown in Fig. 1. The core element is a Nikon Inverted Eclipse Ti fluorescence microscope (Nikon Instruments Inc.). One side port is occupied by the C1 confocal scanning head (Fig. 1b), modified by the DP-attachment for the high-frequency pixel-by-pixel modulation modes. It is performed by photoelastic modulators (PEM-100, Hinds Instruments), one in the excitation light path, in the laser combining unit, and the other (for the analysis of the emission) in the common light path between the C1 scanning head and the microscope. (Generally, the original structure of the CLSM determines the possible positions of the PEMs (Garab and Pomozi 2013).) The original light guide of the microscope is changed to a polarization maintaining fibre (Thorlabs, P3-488PM-FC-2-PM) (Fig. 1c).

The phase-sensitive detection and the signal processing were carried out using a lock-in amplifier (Signal Recovery 7225) and programmable high-impedance amplifiers (HInstra Instruments Ltd.). For the construction of the DP image, the original Nikon EZ-C1 software was used.

### Re-scan confocal microscope (RCM)

The RCM module (Confocal.nl, Netherlands) is a third-party confocal scanning unit based on the re-scan principle (De Luca et al. 2013), placed between a standard fluorescence microscope and the camera. With our quad-band dichroic mirror, it can use four excitation laser lines (405, 488, 561 and 638 nm). Our system is equipped with a 488- and a 638-nm diode laser (Cobolt 06-MLD series). Setting the laser intensity and modulation parameters, the Cobolt Monitor for MLD software (version: 3.2.3.0) is used (Cobolt AB, Sweden). The sCMOS camera is an Andor Zyla 4.2 PLUS from Oxford Instruments, UK. Images are acquired using the NIS-Elements BR software (version: 4.60.00) provided by Nikon Instruments, Japan.

### Polarization modulation

Polarization states/direction of the polarization of the linearly polarized beam can be easily modified using a half-wave plate (HWP). The modulated polarization for the laser excitation (i.e. vertically and horizontally polarized light) for the subsequent images was generated using a horizontal polarizer (Thorlabs, LPVISE200-A) and a liquid crystal (LC) retarder (Thorlabs, LCC1115-A) placed into the excitation light path at 45° (Fig. 1a). The driver for the crystal was a voltage generator (Thorlabs, LCC25). The presented FDLD images were acquired using this module.

### Software development

For the dual purposes of the software extension (automated image acquisition and image processing), different programming languages were used. The code of the LC controller (“*pRCM Manager*”) was written in C language (using low level hardware control for serial communication, capability for programme–programme interactions) and compiled with the Pelles C 8.00.60 compiler (Orinius 2015) partly based on the code of CellFinder third-party microscope manipulation and user interface software (Steinbach and Kaňa 2016). The minimum system requirement is Windows Vista and it supports Windows 7, 8, and 10 as well. The communication between the controller programme and the LC controller unit uses the USB connection and a USB-serial converter module (built-in component of the LC controller).

The image processor application was developed using the Application Designer module of Matlab (MathWorks Matlab, version: R2017a) and the standalone programme was generated using the Application Compiler module. With the help of the Bio-Formats toolbox (Goldberg et al. 2005; Linkert et al. 2010; The Open Microscopy Environment 2018), the ND2 format Nikon images were readable without using the export function of the NIS-Elements software.

### Sample preparation

The used test sample was a *Ginkgo biloba* stem, with Etzold staining (Astra Blue, Fuchsin and Chrysoidine) (Mulisch and Welsch 2015) purchased from MSMedia (Medical & Science Media, Australia).

## Results and discussion

### Modification of the RCM

**Fig. 2:**
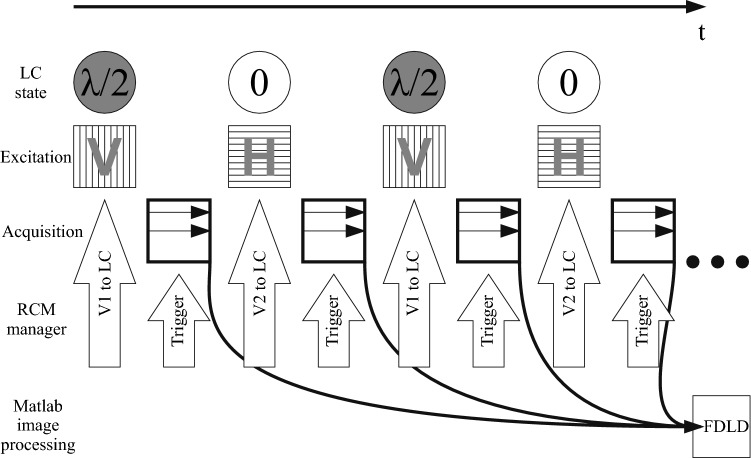
During the imaging series, the *pRCM Manager* software adjusts the voltage level to the LC according to the required polarization state [vertical (V) and horizontal (H) in a pre-set cycle]. The start of each image scan is also triggered by the programme, synchronized to the LC state. The acquired images are processed in Matlab routine

The hardware elements of the DP attachment for the modulation of the polarization state of the laser beam were placed in the RCM module using a custom-designed steel holder, keeping the horizontal polarizer and the liquid crystal retarder head in the excitation light path. Electronic connection for the head was provided by the RCM unit using one of the spare SMA high-frequency coaxial connectors of the RCM laser control panel. In this way, the integrity of the box was retained and no moving element was placed into the RCM unit. Additional advantage of the usage of the LC is the efficient polarization conversion: the transmitted light of the system decreased only by 8% compared to the non-modified version (see Supplementary Figure 2). For adjusting the laser power, instead of the original software solution integrated into NIS-Elements, we used the Cobolt control software that sets the laser intensity on a precise fine scale. The *pRCM Manager* software (see Supplementary Figure 3) developed in our laboratory for this set-up sends the proper voltages to the LC controller unit according to the actual laser wavelength and the required vertical and horizontal polarization states (Fig. 2). The image acquisition of the NIS-Elements software is also triggered by the *pRCM Manager*. For the proper operation, the window position of the NIS-Elements software on the screen is required—this is to be provided after every start (but not before every measurement).

### FDLD images

For FDLD imaging, we used the 488-nm laser excitation and a quad-band dichroic mirror for the available laser lines (405, 488, 561 and 638 nm). To avoid the depolarization effect of the high numerical aperture objectives (Shribak et al. 2002), we applied a 40×/0.60 air objective. In the *pRCM Manager,* the number of the cycles (i.e. image-pair acquisitions using vertically and horizontally polarized excitation) can be adjusted. Accordingly, the acquisitions are triggered by the *pRCM Manager* programme’s timer synchronized to the scanning time. (The RCM unit has three predefined measuring modes: the frame time can be 1, 2 or 4 s—depending on the actual picture size. The dwell time or the image size cannot be adjusted independently.) To ensure the proper overlapping of the sequentially recorded images with different polarizations, fixed samples are required.

The Matlab image processing routine loads the image series and sums up the corresponding items (up to 16 cycles—the raw images are stored in 12 bits and the calculation is based on 16 bit arrays). First, the FDLD values are calculated using the pixel intensity values from the fluorescence images having vertically (V) and horizontally (H) polarized excitation:

$${\text{FDLD}} = \frac{{I_{\text{V}} - I_{\text{H}} }}{{I_{\text{V}} + I_{\text{H}} }}.$$

Then, according to the user-defined threshold, a masking procedure excludes from the FDLD image the pixels having low fluorescence intensity. For the presentation of the fluorescence intensity and the FDLD values, both images are scaled to the best fit: the fluorescence image to the maximum pixel intensity, the FDLD image to the given sensitivity range. For the FDLD representation, a false-colour scale is applied: the horizontally ordered structures are in blue, the vertically ordered ones are in yellow and the pixels not showing any absorbance preference are in grey; the masked areas are in black (see Supplementary Figure 4).

**Fig. 3:**
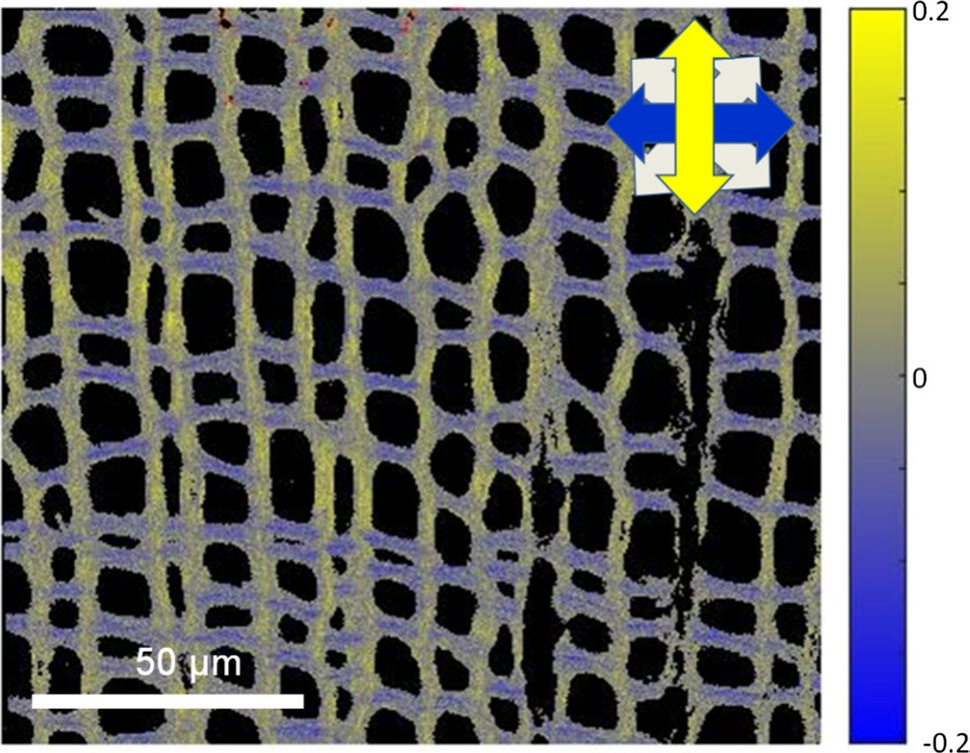
FDLD image of Etzold-stained *Ginkgo biloba* tissue recorded using our DP-equipped RCM (Fig. 1a). The vertically and horizontally excited acquisitions are summed up and the calculated FDLD values are displayed in false colour. The FDLD scale runs in this case from − 0.2 to 0.2

The obtained FDLD image (Fig. 3) proves that the RCM-based DP imaging provides a new and versatile tool for the observation of the cell wall structure. The efficient decomposition and separation technologies, such as those for the biofuel and biomaterial industry, can be based on such structural information.

The further direction of the hardware and software development is to extend the measurable polarization parameters, preferably including all the Mueller matrix elements. The technical opportunity is given for a more complex polarization state generator and polarization state analyser: the RCM unit provides both place and electrical connections for the new optical modulators (Mazumder et al. 2017).

## Electronic supplementary material

Below is the link to the electronic supplementary material.

Supplementary material 1 (PDF 200 kb)

**Supplementary Figure** **1** Test slide measured using the C1 confocal head (and having a PMT detector) under different conditions and using the RCM (having an Andor Zyla 4.2 PLUS sCMOS camera). 50/60 μm pinhole sizes and 0.495/0.5 fps imaging rates were the closest available values. (TIFF 193 kb)

**Supplementary Figure** **2** The efficiency of the light transmission of the system (imaging the same multidirectional sample) for the non-modified setup and for the DP extension. (TIFF 520 kb)

**Supplementary Figure** **3** The user interface of the *pRCM Manager* software. It controls the LC and the synchronised imaging as well. (TIFF 383 kb)

**Supplementary Figure** **4** The Matlab image processing routine for conversion and FDLD calculation. (TIFF 824 kb)
