# Supplementary material for: Fluorescence-detected linear dichroism imaging in a re-scan confocal microscope equipped with differential polarization attachment
Source: Eur Biophys J. 2019 Apr 13;48(5):457–63. doi: 10.1007/s00249-019-01365-4 (PMC6647120; doi:10.1007/s00249-019-01365-4)
Supplement: Supplementary file 1 — Supplementary material 1 (PDF 200 kb) [file 249_2019_1365_MOESM1_ESM.pdf]

```
#define WIN32_LEAN_AND_MEAN
/* #define NOCRYPT */
/* #define NOSERVICE */
/* #define NOMCX */
/* #define NOIME */

#include <windows.h>
#include <windowsx.h>
#include <commctrl.h>
#include <tchar.h>
#include <stdlib.h>
#include <stdio.h>
#include "main.h"

#define NELEMS(a)  (sizeof(a) / sizeof((a)[0]))

/** Prototypes **************************************************************/

static INT_PTR CALLBACK MainDlgProc(HWND, UINT, WPARAM, LPARAM);
static LRESULT WINAPI AboutDlgProc(HWND, UINT, WPARAM, LPARAM);
static LRESULT WINAPI RunDlgProc(HWND, UINT, WPARAM, LPARAM);
static LRESULT WINAPI SerparDlgProc(HWND, UINT, WPARAM, LPARAM);
int Click(int, int);
int setcom(HANDLE);

/** Global variables ********************************************************/
```

```
static HANDLE ghInstance;

char
ports[9][10]={"\\\\.\\COM1","\\\\.\\COM2","\\\\.\\COM3","\\\\.\\COM4","\\\\.\\COM5","\\\\.\\CO
M6","\\\\.\\COM7","\\\\.\\COM8","\\\\.\\COM9"};

int currport=-1;

int currcommenu=0;

int currlaser=-1;

float V1[]={2.1,2.2,2.3,2.4,2.5,2.6,2.7,2.8};

float V2[]={7.1,7.2,7.3,7.4,7.5,7.6,7.7,7.8};

int currcyc=0;

HANDLE handlePort=INVALID_HANDLE_VALUE;

HMENU hMenu;

HWND mainhwndDlg;

int cap=0; // egerkoordinatahoz

HWND list_log=NULL;


int PASCAL WinMain(HINSTANCE hInstance, HINSTANCE hPrevInstance, LPSTR lpszCmdLine, int
nCmdShow)

{

    INITCOMMONCONTROLSEX icc;

    WNDCLASSEX wcx;


    ghInstance = hInstance;


    /* Initialize common controls. Also needed for MANIFEST's */

    /*

     * TODO: set the ICC_???_CLASSES that you need.

     */
```

```
    icc.dwSize = sizeof(icc);
    icc.dwICC = ICC_WIN95_CLASSES
/*|ICC_COOL_CLASSES|ICC_DATE_CLASSES|ICC_PAGESCROLLER_CLASS|ICC_USEREX_CLASSES|... */;
    InitCommonControlsEx(&icc);

    /* Load Rich Edit control support */
    /*
     * TODO: uncomment one of the lines below, if you are using a Rich Edit control.
     */
    // LoadLibrary(_T("riched32.dll"));  // Rich Edit v1.0
    // LoadLibrary(_T("riched20.dll"));  // Rich Edit v2.0, v3.0

    /*
     * TODO: uncomment line below, if you are using the Network Address control (Windows Vista+).
     */
    // InitNetworkAddressControl();

    /* Get system dialog information */
    wcx.cbSize = sizeof(wcx);
    if (!GetClassInfoEx(NULL, MAKEINTRESOURCE(32770), &wcx))
        return 0;

    /* Add our own stuff */
    wcx.hInstance = hInstance;
    wcx.hIcon = LoadIcon(hInstance, MAKEINTRESOURCE(IDR_ICO_MAIN));
    wcx.lpszMenuName = MAKEINTRESOURCE(IDR_MNU_MAIN);
    wcx.lpszClassName = _T("LC_FDLDClass");
    if (!RegisterClassEx(&wcx))
```

```
        return 0;

    /* The user interface is a modal dialog box */
    return DialogBox(hInstance, MAKEINTRESOURCE(DLG_MAIN), NULL, (DLGPROC)MainDlgProc);
}



static INT_PTR CALLBACK MainDlgProc(HWND hwndDlg, UINT uMsg, WPARAM wParam, LPARAM
lParam)
{
        char dummy[500];
        int i;
        POINT xy;
        unsigned long int length;

    switch (uMsg)
    {
        case WM_INITDIALOG: {
                        hMenu=GetMenu(hwndDlg);
                        mainhwndDlg=hwndDlg;
                        SetDlgItemInt(hwndDlg,ID_E_XC,0,TRUE);
                        SetDlgItemInt(hwndDlg,ID_E_YC,0,TRUE);
                        //default values
                        SetDlgItemInt(hwndDlg,ID_E_CYC,5,TRUE);
                        SetDlgItemInt(hwndDlg,ID_E_INT,3500,TRUE);
                        CheckRadioButton(hwndDlg,ID_C_405,ID_C_1152,ID_C_488);
                        currlaser=2;
                        sprintf(dummy,"%.1f",V1[currlaser]);
```

```
                        SetDlgItemText(hwndDlg,ID_E_V1,dummy);
                        sprintf(dummy,"%.1f",V2[currlaser]);
                        SetDlgItemText(hwndDlg,ID_E_V2,dummy);
                        currcommenu=IDM_COM5;
                        currport=currcommenu-IDM_COM1;
                        CheckMenuItem(hMenu, currcommenu, MF_BYCOMMAND | MF_CHECKED);


                        SetDlgItemText(hwndDlg,ID_S_STATUS,"Welcome!");
   return TRUE;
                }
case WM_SIZE:


   return TRUE;
case WM_LBUTTONUP: {
                        if(cap)
                        {
                                ReleaseCapture();
                                xy.x = GET_X_LPARAM(lParam);
                                xy.y = GET_Y_LPARAM(lParam);
                                ClientToScreen(hwndDlg,&xy);
                                SetDlgItemInt(hwndDlg,ID_E_XC,xy.x,TRUE);
                                SetDlgItemInt(hwndDlg,ID_E_YC,xy.y,TRUE);
                                SetDlgItemText(hwndDlg,ID_S_STATUS,"Captured");
                                cap=0;
                        }
                        return TRUE;
                }
```

```
case WM_COMMAND:
  switch (GET_WM_COMMAND_ID(wParam, lParam))
  {
                        case ID_C_405:
                        case ID_C_458:
                        case ID_C_488:
                        case ID_C_514:
                        case ID_C_543:
                        case ID_C_594:
                        case ID_C_633:
                        case ID_C_1152:
                                for(i=ID_C_405;i<=ID_C_1152;i++)
                                        if(IsDlgButtonChecked(hwndDlg,i)==BST_CHECKED) {
                                                currlaser=i-ID_C_405;

                                                sprintf(dummy,"%.1f",V1[currlaser]);
                                                SetDlgItemText(hwndDlg,ID_E_V1,dummy);
                                                sprintf(dummy,"%.1f",V2[currlaser]);
                                                SetDlgItemText(hwndDlg,ID_E_V2,dummy);
                                                break;
                                }
                                return TRUE;
    case ID_B_GETC: {
                                SetCapture(hwndDlg);
      cap=1;
                                SetDlgItemInt(hwndDlg,ID_E_XC,-42,TRUE);
                                SetDlgItemInt(hwndDlg,ID_E_YC,-42,TRUE);
```

```
                                        SetDlgItemText(hwndDlg,ID_S_STATUS,"Press L_Button here and relase where you need the ACQ click.");
                return TRUE;
                                }
                                case ID_B_START: {
                                        if(GetDlgItemInt(mainhwndDlg,ID_E_INT,NULL,TRUE)<200 || GetDlgItemInt(mainhwndDlg,ID_E_INT,NULL,TRUE)>10000) {
                                                SetDlgItemInt(mainhwndDlg,ID_E_INT,2500,TRUE);
                                                i=MessageBox(hwndDlg,"Iteration time was out of range - corrected\nDo you want to continue?","Warning", MB_YESNO | MB_ICONWARNING | MB_DEFBUTTON1);
                                        }
                                        if(i==IDNO)
                                                return TRUE;
                                        SetDlgItemText(hwndDlg,ID_S_STATUS,"Running...");
                                        DialogBox(ghInstance, MAKEINTRESOURCE(DLG_RUN), hwndDlg, (DLGPROC)RunDlgProc);
                                        //for(i=IDM_COM1;i<=IDM_COM9;i++)
                                        //      EnableMenuItem(hMenu, i, MF_BYCOMMAND | MF_DISABLED);


                                        return TRUE;
                                }
                                //case ID_B_BREAK:
                                //      return TRUE;
                                //case IDOK:
                                //      return TRUE;
                                case IDM_COM1:
                                case IDM_COM2:
            case IDM_COM3:
```

```
case IDM_COM4:
case IDM_COM5:
case IDM_COM6:
case IDM_COM7:
case IDM_COM8:
case IDM_COM9:
                    if(currcommenu)
                        CheckMenuItem(hMenu, currcommenu,
MF_BYCOMMAND | MF_UNCHECKED);
                    currcommenu=GET_WM_COMMAND_ID(wParam, lParam);
                    currport=currcommenu-IDM_COM1;
                    //SetDlgItemText(hwndDlg,ID_E_CYC,ports[currport]);
                    //SetDlgItemInt(hwndDlg,ID_E_XC,currcommenu,TRUE);
                    //SetDlgItemInt(hwndDlg,ID_E_YC,currport,TRUE);
                    CheckMenuItem(hMenu, currcommenu, MF_BYCOMMAND |
MF_CHECKED);
  return TRUE;
                case IDM_ABOUT:
                    DialogBox(ghInstance, MAKEINTRESOURCE(DLG_ABOUT),
hwndDlg, (DLGPROC)AboutDlgProc);
                    return TRUE;
                case IDM_CONNECT: {
                    if(handlePort==INVALID_HANDLE_VALUE) {
                        handlePort=CreateFile(ports[currport],
                                GENERIC_READ | GENERIC_WRITE,
// Specify mode that open device.
                                0,              // the devide
isn't shared.
                                NULL,           // the object
gets a default security.
```

```
					OPEN_EXISTING,			//
Specify which action to take on file.
					0,				// default.
					NULL);				// default.
				if(handlePort==INVALID_HANDLE_VALUE){
					MessageBox(hwndDlg,"Opening COM port
failed!\nTry an other one.", "COM Error", MB_OK |MB_ICONEXCLAMATION);

	SetDlgItemText(hwndDlg,ID_S_STATUS,"Opening COM port failed.");
					return TRUE;
				}
				// #### test device: kerdes-valasz azonositas...
				if(setcom(handlePort)==-1) {
					SetDlgItemText(hwndDlg,ID_S_STATUS,"Port
initialization failed");
					if(CloseHandle(handlePort) == 0)
						MessageBox(hwndDlg,"Closing COM
port failed!\n?!?!?!", "COM Error", MB_OK |MB_ICONEXCLAMATION);
					handlePort=INVALID_HANDLE_VALUE;
					return TRUE;
				}
				CheckMenuItem(hMenu, IDM_CONNECT,
MF_BYCOMMAND | MF_CHECKED);
				SetDlgItemText(hwndDlg,ID_S_STATUS,"Port
opened");
				if (WriteFile(handlePort,
						"enable=1\r",
						9,
					&length,NULL)==0)	{
					sprintf(dummy,"write error:
%d",GetLastError());
```

```
SetDlgItemText(hwndDlg,ID_S_STATUS,dummy);
                    }
                }
                else {
                    if(CloseHandle(handlePort) == 0)
                        MessageBox(hwndDlg,"Closing COM port
failed!\n?!?!?!", "COM Error", MB_OK |MB_ICONEXCLAMATION);
                    handlePort=INVALID_HANDLE_VALUE;
                    CheckMenuItem(hMenu, IDM_CONNECT,
MF_BYCOMMAND | MF_UNCHECKED);
                    SetDlgItemText(hwndDlg,ID_S_STATUS,"Port
closed");
                }
                return TRUE;
            }
    case IDM_EXIT:
      EndDialog(hwndDlg, TRUE);
      return TRUE;
    case IDM_42HZ:
                if(handlePort==INVALID_HANDLE_VALUE) {
                    SetDlgItemText(hwndDlg,ID_S_STATUS,"Port not
available!");
                    return TRUE;
                }
                if (WriteFile(handlePort,
                        "freq=42\r",
                        8,
                    &length,NULL)==0)       {
```

```
                        sprintf(dummy,"write error: %d",GetLastError());
                        SetDlgItemText(hwndDlg,ID_S_STATUS,dummy);
                        return TRUE;
                    }
        return TRUE;
    case IDM_SERPAR:
                    CreateDialog(ghInstance, MAKEINTRESOURCE(DLG_SERPAR),
hwndDlg, (DLGPROC)SerparDlgProc);
        return TRUE;
    case IDM_MODE1:
                    if(handlePort==INVALID_HANDLE_VALUE) {
                        SetDlgItemText(hwndDlg,ID_S_STATUS,"Port not
available!");
                        return TRUE;
                    }
                    if (WriteFile(handlePort,
                            "mode=1\r",
                            7,
                        &length,NULL)==0)       {
                        sprintf(dummy,"write error: %d",GetLastError());
                        SetDlgItemText(hwndDlg,ID_S_STATUS,dummy);
                        return TRUE;
                    }
        return TRUE;
    case IDM_MODE2:
                    if(handlePort==INVALID_HANDLE_VALUE) {
                        SetDlgItemText(hwndDlg,ID_S_STATUS,"Port not
available!");
                        return TRUE;
```

```
                                                }
                                                if (WriteFile(handlePort,
                                                              "mode=2\r",
                                                              7,
                                                       &length,NULL)==0)       {
                                                       sprintf(dummy,"write error: %d",GetLastError());
                                                       SetDlgItemText(hwndDlg,ID_S_STATUS,dummy);
                                                       return TRUE;
                                                }
                return TRUE;
            }
            break;

        case WM_CLOSE:
            EndDialog(hwndDlg, 0);
            return TRUE;

        /*
         * TODO: Add more messages, when needed.
         */
    }

    return FALSE;
}

static LRESULT CALLBACK AboutDlgProc(HWND hDlg, UINT uMsg, WPARAM wParam, LPARAM
lParam)

{
```

```
    switch (uMsg)
    {
        case WM_INITDIALOG:
            return TRUE;
        case WM_COMMAND:
            switch (wParam)
            {
                case IDOK:
                case IDCANCEL:
                    EndDialog(hDlg, TRUE);
                    return TRUE;
            }
            break;
    }

    return FALSE;
}
static LRESULT CALLBACK SerparDlgProc(HWND hDlg, UINT uMsg, WPARAM wParam, LPARAM
lParam)
{
//          COMMTIMEOUTS timeout;
    COMMCONFIG conf;
            DWORD cs;
            char dummy[100];

    switch (uMsg)
    {
        case WM_INITDIALOG:
```

```
                    list_log=GetDlgItem(hDlg,ID_L_LOG);
        return TRUE;
      case WM_COMMAND:
        switch (wParam)        {
          case ID_B_CHECKPORT:
                                    if(handlePort==INVALID_HANDLE_VALUE) {
                                        handlePort=CreateFile(ports[currport],
                                                        GENERIC_READ | GENERIC_WRITE,
// Specify mode that open device.
                                                        0,                    // the devide
isn't shared.
                                                        NULL,                   // the object
gets a default security.
                                                        OPEN_EXISTING,              //
Specify which action to take on file.
                                                        0,                    // default.
                                                        NULL);                  // default.
                                        if(handlePort==INVALID_HANDLE_VALUE){
                                            ListBox_AddString(list_log,"*** Opening COM
port failed! Try an other one.");

        ListBox_SetCaretIndex(list_log,ListBox_GetCount(list_log));
                                            return TRUE;
                                        }
                                    }

                                    cs=sizeof(conf);
                                    GetCommConfig(handlePort,&conf,&cs);

                                    sprintf(dummy,"conf.dwSize %d",sizeof(COMMCONFIG));
```

```
ListBox_AddString(list_log,dummy);
sprintf(dummy,"conf.wVersion %d",conf.wVersion);
ListBox_AddString(list_log,dummy);

sprintf(dummy,"conf.dcb.DCBlength %d",sizeof(DCB));
ListBox_AddString(list_log,dummy);
sprintf(dummy,"conf.dcb.BaudRate %d",conf.dcb.BaudRate);
ListBox_AddString(list_log,dummy);
sprintf(dummy,"conf.dcb.fBinary %d",conf.dcb.fBinary);
ListBox_AddString(list_log,dummy);
sprintf(dummy,"conf.dcb.fParity %d",conf.dcb.fParity);
ListBox_AddString(list_log,dummy);
sprintf(dummy,"conf.dcb.fOutxCtsFlow
%d",conf.dcb.fOutxCtsFlow);
ListBox_AddString(list_log,dummy);
sprintf(dummy,"conf.dcb.fOutxDsrFlow
%d",conf.dcb.fOutxDsrFlow);
ListBox_AddString(list_log,dummy);
sprintf(dummy,"conf.dcb.fDtrControl %d,
DTR_CONTROL_DISABLE? %d",conf.dcb.fDtrControl,conf.dcb.fDtrControl==DTR_CONTROL_DISABLE);
ListBox_AddString(list_log,dummy);
sprintf(dummy,"conf.dcb.fDsrSensitivity
%d",conf.dcb.fDsrSensitivity);
ListBox_AddString(list_log,dummy);
sprintf(dummy,"conf.dcb.fTXContinueOnXoff
%d",conf.dcb.fTXContinueOnXoff);
ListBox_AddString(list_log,dummy);
sprintf(dummy,"conf.dcb.fOutX %d",conf.dcb.fOutX);
ListBox_AddString(list_log,dummy);
```

```
sprintf(dummy,"conf.dcb.fInX %d",conf.dcb.fInX);
ListBox_AddString(list_log,dummy);
sprintf(dummy,"conf.dcb.fErrorChar %d",conf.dcb.fErrorChar);
ListBox_AddString(list_log,dummy);
sprintf(dummy,"conf.dcb.fNull %d",conf.dcb.fNull);
ListBox_AddString(list_log,dummy);
sprintf(dummy,"conf.dcb.fRtsControl %d, RTS_CONTROL_DISABLE? %d",conf.dcb.fRtsControl,conf.dcb.fRtsControl==RTS_CONTROL_DISABLE);
ListBox_AddString(list_log,dummy);
sprintf(dummy,"conf.dcb.fAbortOnError %d",conf.dcb.fAbortOnError);
ListBox_AddString(list_log,dummy);
sprintf(dummy,"conf.dcb.XonLim %d",conf.dcb.XonLim);
ListBox_AddString(list_log,dummy);
sprintf(dummy,"conf.dcb.XoffLim %d",conf.dcb.XoffLim);
ListBox_AddString(list_log,dummy);
sprintf(dummy,"conf.dcb.ByteSize %d",conf.dcb.ByteSize);
ListBox_AddString(list_log,dummy);
sprintf(dummy,"conf.dcb.Parity %d, NOPARITY? %d",conf.dcb.Parity,conf.dcb.Parity==NOPARITY);
ListBox_AddString(list_log,dummy);
sprintf(dummy,"conf.dcb.StopBits %d, ONESTOPBIT? %d",conf.dcb.StopBits,conf.dcb.StopBits==ONESTOPBIT);
ListBox_AddString(list_log,dummy);
sprintf(dummy,"conf.dcb.XonChar %d",conf.dcb.XonChar);
ListBox_AddString(list_log,dummy);
sprintf(dummy,"conf.dcb.XoffChar %d",conf.dcb.XoffChar);
ListBox_AddString(list_log,dummy);
```

```
                                        sprintf(dummy,"conf.dcb.ErrorChar %d",conf.dcb.ErrorChar);
                                        ListBox_AddString(list_log,dummy);
                                        sprintf(dummy,"conf.dcb.EofChar %d",conf.dcb.EofChar);
                                        ListBox_AddString(list_log,dummy);
                                        sprintf(dummy,"conf.dcb.EvtChar %d",conf.dcb.EvtChar);
                                        ListBox_AddString(list_log,dummy);
                                        ListBox_SetCaretIndex(list_log,ListBox_GetCount(list_log));



                                        if(CloseHandle(handlePort) == 0) {
                                              ListBox_AddString(list_log,"*** Closing port
failed!?!");

      ListBox_SetCaretIndex(list_log,ListBox_GetCount(list_log));
                                        }
                                        handlePort=INVALID_HANDLE_VALUE;
                                        return TRUE;
       case IDCANCEL:
         EndDialog(hDlg, TRUE);
         return TRUE;
     }
     break;
  }

  return FALSE;
}
static LRESULT CALLBACK RunDlgProc(HWND hDlg, UINT uMsg, WPARAM wParam, LPARAM lParam)
{
```

```
            char dummy[100];
            unsigned long int length;

    switch (uMsg)
    {
      case WM_INITDIALOG:
                                if(handlePort==INVALID_HANDLE_VALUE) {
                                        SetDlgItemText(mainhwndDlg,ID_S_STATUS,"No COM connection!");
             EndDialog(hDlg, TRUE);
                                }
                                currcyc=0;

            SendMessage(GetDlgItem(hDlg,ID_P_PROG),PBM_SETRANGE,0,0+256*256*GetDlgItemInt(mainhwndDlg,ID_E_CYC,NULL,FALSE)*2);
                                Button_Enable(GetDlgItem(hDlg,ID_B_CLOSE),FALSE);
                                SetDlgItemText(hDlg,ID_S_PROG,"Init...");
//                              sprintf(dummy,"volt1=%.1f\r",V1[currlaser]);
//                              sprintf(dummy,"volt2=%.1f\r",V2[currlaser]);
// ### Write file - elkuldeni az aktualis feszultsegertekeket
                                SetTimer(hDlg,42,5000,NULL);
          return TRUE;
      case WM_TIMER: {
                                KillTimer(hDlg,42);
                                currcyc++;
                                SendMessage(GetDlgItem(hDlg,ID_P_PROG),PBM_SETPOS,currcyc,0);

            sprintf(dummy,"%d/%d",currcyc,GetDlgItemInt(mainhwndDlg,ID_E_CYC,NULL,FALSE)*2);
                                SetDlgItemText(hDlg,ID_S_PROG,dummy);
```

```
				if(currcyc%2) { // paratlan kor
					sprintf(dummy,"mode=1\r");
				}
				else { // paros kor
					sprintf(dummy,"mode=2\r");
				}
				//SetDlgItemText(hDlg,ID_S_PROG,dummy);
				if (WriteFile(handlePort,
						dummy,
						7,
					&length,NULL)==0)	{
					sprintf(dummy,"write error: %d",GetLastError());
					MessageBox(hDlg,dummy,"Write
error",MB_OK|MB_ICONEXCLAMATION);
					SetDlgItemText(mainhwndDlg,ID_S_STATUS,"Write error
occoured.");
         EndDialog(hDlg, TRUE);

				}
				if(IsDlgButtonChecked(mainhwndDlg,ID_C_ONE)==BST_UNCHECKED ||
currcyc==1)

	Click(GetDlgItemInt(mainhwndDlg,ID_E_XC,NULL,TRUE),GetDlgItemInt(mainhwndDlg,ID_E_Y
C,NULL,TRUE));
				if(currcyc<(int)GetDlgItemInt(mainhwndDlg,ID_E_CYC,NULL,FALSE)*2)

	SetTimer(hDlg,42,GetDlgItemInt(mainhwndDlg,ID_E_INT,NULL,TRUE),NULL);
				else {
					Button_Enable(GetDlgItem(hDlg,ID_B_CLOSE),TRUE);
					Button_Enable(GetDlgItem(hDlg,ID_B_BREAK),FALSE);
```

```
                            }

        return TRUE;
                    }
      case WM_COMMAND:
        switch (wParam)
        {
          case IDOK:
          case ID_B_BREAK:
                                        SetDlgItemText(mainhwndDlg,ID_S_STATUS,"Stopped!");
            EndDialog(hDlg, TRUE);
            return TRUE;
          case ID_B_CLOSE:
                                        SetDlgItemText(mainhwndDlg,ID_S_STATUS,"Finished.");
            EndDialog(hDlg, TRUE);
            return TRUE;
        }
        break;
    }

    return FALSE;
}

int Click(int x, int y)
{
         INPUT a[5];
         int i=0;
```

```
long mx,my;

mx=x*65535/GetSystemMetrics(SM_CXVIRTUALSCREEN);
my=y*65535/GetSystemMetrics(SM_CYVIRTUALSCREEN);

a[i].type=INPUT_MOUSE;
a[i].mi.dx=mx;
a[i].mi.dy=my;
a[i].mi.mouseData=0;
a[i].mi.dwFlags=MOUSEEVENTF_ABSOLUTE|MOUSEEVENTF_MOVE|MOUSEEVENTF_VIRTUALDESK;
a[i].mi.time=0;
a[i++].mi.dwExtraInfo=GetMessageExtraInfo();

a[i].type=INPUT_MOUSE;
a[i].mi.dx=0;
a[i].mi.dy=0;
a[i].mi.mouseData=0;
a[i].mi.dwFlags=MOUSEEVENTF_LEFTDOWN;
a[i].mi.time=0;
a[i++].mi.dwExtraInfo=GetMessageExtraInfo();

a[i].type=INPUT_MOUSE;
a[i].mi.dx=0;
a[i].mi.dy=0;
a[i].mi.mouseData=0;
a[i].mi.dwFlags=MOUSEEVENTF_LEFTUP;
a[i].mi.time=0;
```

```
		a[i++].mi.dwExtraInfo=GetMessageExtraInfo();

		SendInput(i,a,sizeof(a[0]));
		//MessageBeep(MB_OK);
		return 0;
}

int setcom(HANDLE h)
{
		COMMTIMEOUTS timeout;
    COMMCONFIG conf;
		DWORD cs;

		cs=sizeof(conf);
		GetCommConfig(h,&conf,&cs);

		conf.dwSize=sizeof(COMMCONFIG);
		conf.dcb.DCBlength=sizeof(DCB);
		conf.dcb.BaudRate=CBR_115200;
		conf.dcb.fBinary=TRUE;
		conf.dcb.fParity=FALSE;
		conf.dcb.fOutxCtsFlow=FALSE;
		conf.dcb.fOutxCtsFlow=FALSE;
		conf.dcb.fOutxDsrFlow=FALSE;
//		conf.dcb.fDtrControl=DTR_CONTROL_DISABLE ;
		conf.dcb.fDsrSensitivity=FALSE;
		conf.dcb.fTXContinueOnXoff=TRUE;
```

```
	conf.dcb.fOutX=FALSE;
	conf.dcb.fInX=FALSE;
	conf.dcb.fErrorChar=FALSE;
	conf.dcb.fNull=FALSE;
//	conf.dcb.fRtsControl=RTS_CONTROL_DISABLE;
	conf.dcb.fAbortOnError=FALSE;
	conf.dcb.wReserved=0;
	conf.dcb.XonLim=2048;
	conf.dcb.XoffLim=512;
	conf.dcb.ByteSize=8;
	conf.dcb.Parity=NOPARITY;
	conf.dcb.StopBits=ONESTOPBIT;
	conf.dcb.XonChar=17;
	conf.dcb.XoffChar=19;
	conf.dcb.ErrorChar=0;
	conf.dcb.EofChar=0;
	conf.dcb.EvtChar=0;

	if(SetCommConfig(h,&conf,cs)==0)
		return -1;

	GetCommTimeouts(handlePort,&timeout);
	timeout.ReadIntervalTimeout=25;
	timeout.ReadTotalTimeoutMultiplier=1;
	timeout.ReadTotalTimeoutConstant=25;
	timeout.WriteTotalTimeoutMultiplier=5;
	timeout.WriteTotalTimeoutConstant=5;
```

```
        SetCommTimeouts(h,&timeout);

        return 0;
}

// INCLUDE FILE generated by "Pelles C for Windows, version 1.00".

#define DLG_MAIN  1001
#define IDR_ICO_MAIN  8001
#define IDR_MNU_MAIN  2001
#define IDM_CONNECT  6001
#define IDM_EXIT  6002
#define IDM_COM1  6003
#define IDM_COM2  6004
#define IDM_COM3  6005
#define IDM_COM4  6006
#define IDM_COM5  6007
#define IDM_COM6  6008
#define IDM_COM7  6009
#define IDM_COM8  6010
#define IDM_COM9  6011
#define IDM_ABOUT  6012
#define DLG_ABOUT  1002
#define ID_C_405  4001
#define ID_C_458  4002
#define ID_C_488  4003
#define ID_C_514  4004
```

```
#define ID_C_543  4005
#define ID_C_594  4006
#define ID_C_633  4007
#define ID_C_1152  4008
#define ID_E_XC  4015
#define ID_E_YC  4016
#define ID_B_GETC  4017
#define ID_B_START  4019
#define ID_B_BREAK  4020
#define ID_E_CYC  4011
#define ID_S_STATUS  4021
#define ID_E_V1  4024
#define ID_E_V2  4025
#define ID_S_CYC  4026
#define DLG_RUN  1003
#define ID_P_PROG  4009
#define ID_S_PROG  4010
#define ID_B_CLOSE  4012
#define IDM_TESTLCCOM  6013
#define IDM_MODE1  6014
#define IDM_MODE2  6015
#define IDM_42HZ  6016
#define ID_E_INT  4028
#define DLG_SERPAR  1004
#define ID_B_CHECKPORT  4014
#define IDM_SERPAR  6017
#define ID_L_LOG  4013
```

```
#define ID_C_ONE  4029
```
