# Supplementary figures and images for: Fluorescence-detected linear dichroism imaging in a re-scan confocal microscope equipped with differential polarization attachment
Source: Eur Biophys J. 2019 Apr 13;48(5):457–63. doi: 10.1007/s00249-019-01365-4 (PMC6647120; doi:10.1007/s00249-019-01365-4)

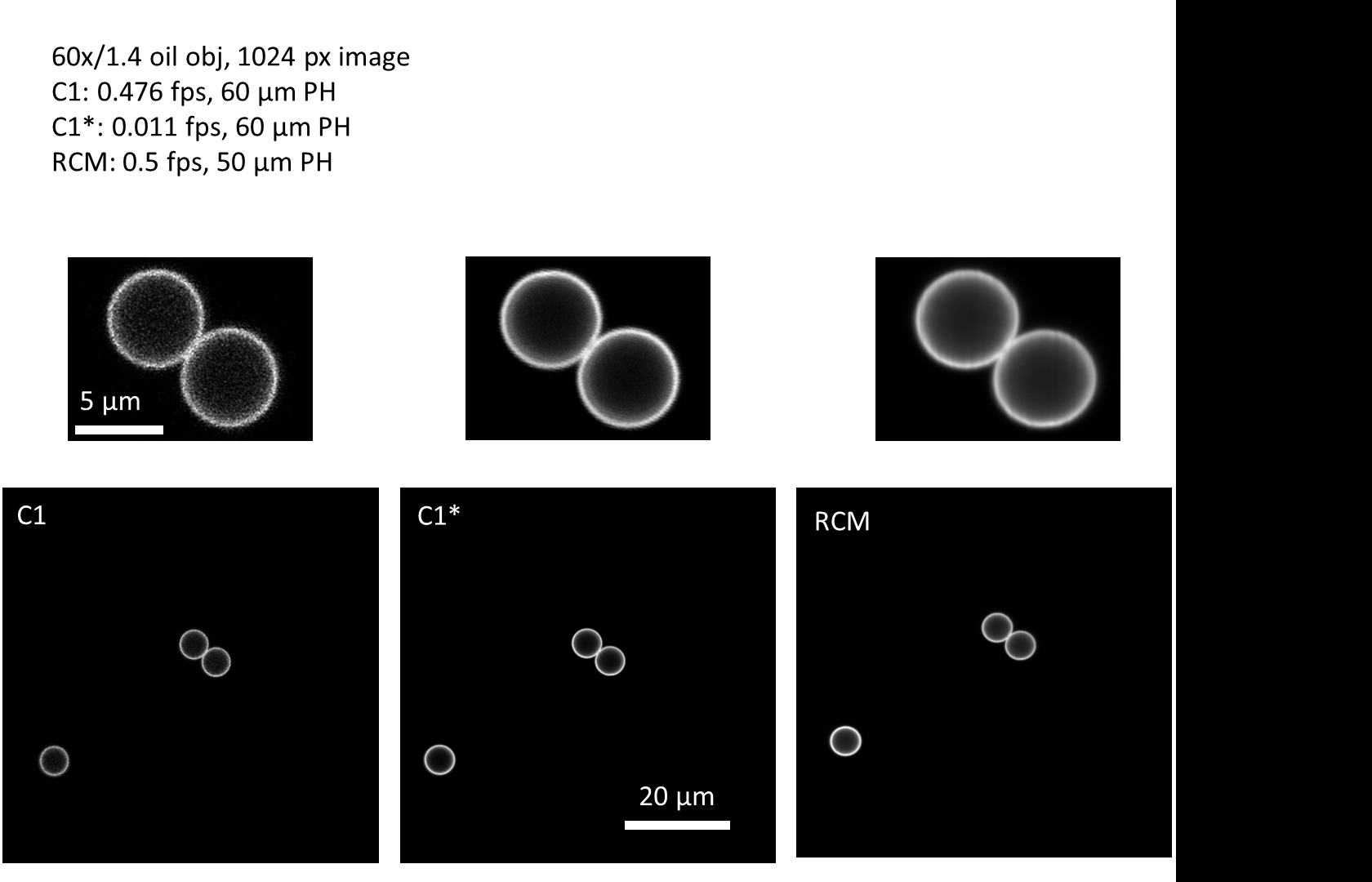

Supplement: Supplementary file 2 — Supplementary Figure 1 Test slide measured using the C1 confocal head (and having a PMT detector) under different conditions and using the RCM (having an Andor Zyla 4.2 PLUS sCMOS camera). 50/60 μm pinhole sizes and 0.495/0.5 fps imaging rates were the closest available values. (TIFF 193 kb) [file 249_2019_1365_MOESM2_ESM.tif]

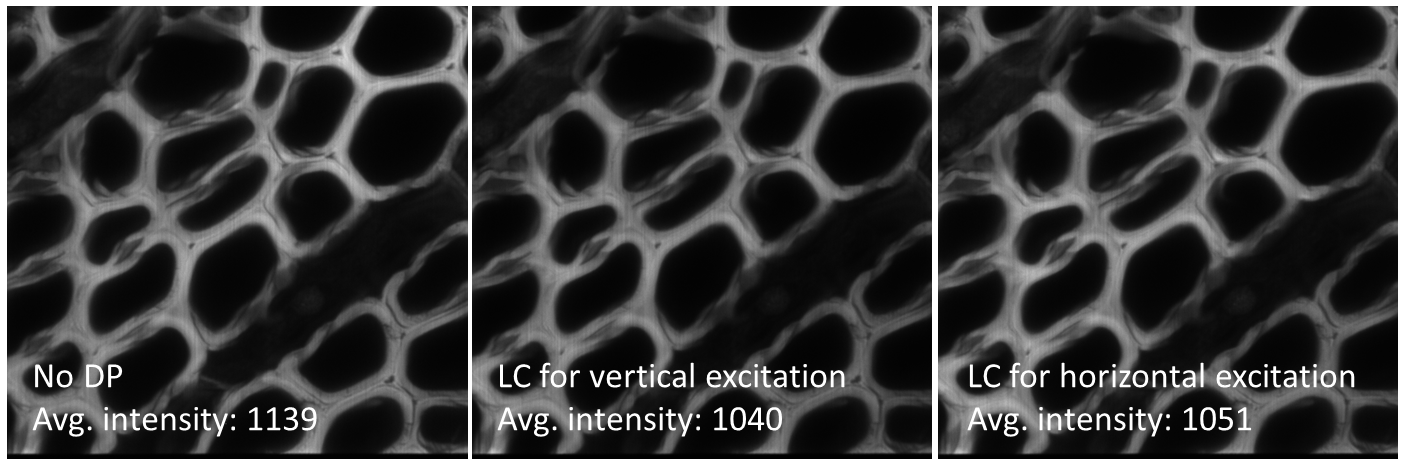

Supplement: Supplementary file 3 — Supplementary Figure 2 The efficiency of the light transmission of the system (imaging the same multidirectional sample) for the non-modified setup and for the DP extension. (TIFF 520 kb) [file 249_2019_1365_MOESM3_ESM.tif]

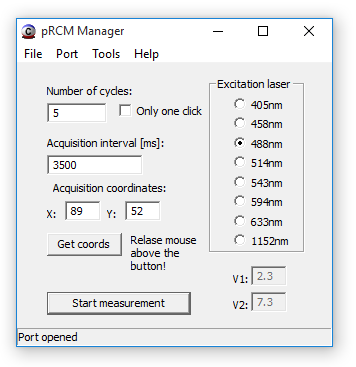

Supplement: Supplementary file 4 — Supplementary Figure 3 The user interface of the pRCM Manager software. It controls the LC and the synchronised imaging as well. (TIFF 383 kb) [file 249_2019_1365_MOESM4_ESM.tif]

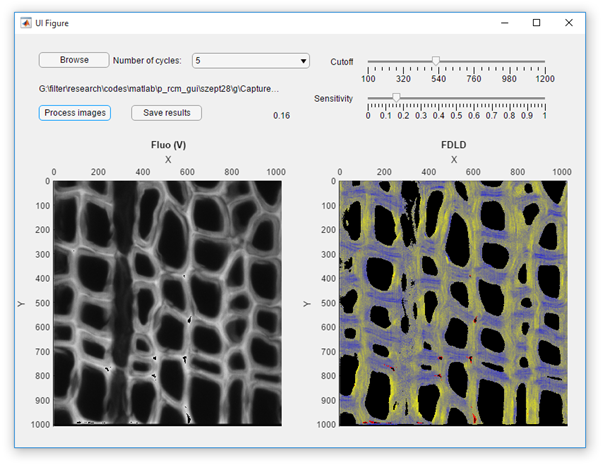

Supplement: Supplementary file 5 — Supplementary Figure 4 The Matlab image processing routine for conversion and FDLD calculation. (TIFF 824 kb) [file 249_2019_1365_MOESM5_ESM.tif]
